# Effect of Fetal Sex on Maternal and Obstetric Outcomes

**DOI:** 10.3389/fped.2017.00144

**Published:** 2017-06-19

**Authors:** Mohammed Al-Qaraghouli, Yu Ming Victor Fang

**Affiliations:** ^1^Department Obstetrics and Gynecology, Division Maternal-Fetal Medicine, UConn Health John Dempsey Hospital, Farmington, CT, United States

**Keywords:** fetal, sex, obstetrical, maternal, outcome

## Abstract

Fetal sex plays an important role in modifying the course and complications related to pregnancy and may also have an impact on maternal health and well-being both during and after pregnancy. The goal of this article is to review and summarize the findings from published research on physiologic and pathologic changes that may be affected by fetal sex and the effect of these changes on the maternal and obstetrical outcomes. This will help create awareness that fetal sex is not just a random chance event but an interactive process between the mother, the placenta, and the fetus. The reported effects of male sex on the course of pregnancy and delivery include higher incidence of preterm labor in singletons and twins, failure of progression in labor, true umbilical cord knots, cord prolapse, nuchal cord, higher cesarean section rate, higher heart rate variability with increased frequency, and duration of decelerations without acidemia and increased risk of gestational diabetes mellitus through the poor beta cells function. Similarly, female fetal sex has been reported to modify pregnancy and delivery outcomes including altered fetal cardiac hemodynamics, increased hypertensive diseases of pregnancy, higher vulnerability of developing type 2 DM after pregnancy possibly because of influences on increased maternal insulin resistance. Placental function is also influenced by fetal sex. Vitamin D metabolism in the placenta varies by fetal sex; and the placenta of a female fetus is more responsive to the relaxing action of magnesium sulfate. Male and female feto-placental units also vary in their responses to environmental toxin exposure. The association of fetal sex with stillbirths is controversial with many studies reporting higher risk of stillbirth in male fetuses; although some smaller and limited studies have reported more stillbirths with female fetus pregnancies. Maternal status such as BMI may in turn also affect the fetus and the placenta in a sex-specific manner. There is probably a sex-specific maternal–placental–fetal interaction that has significant biological implications of which the mechanisms may be genetic, epigenetic, or hormonal. Determination of fetal sex may therefore be an important consideration in management of pregnancy and childbirth.

## Introduction

Human sex is the result of a complex prenatal interaction of genetic, gonadal, and hormonal factors; and establishment of gender involves additional postnatal phenotypic and psychological factors. The prenatal sex-specific interactions between the mother, the placenta, and the fetus have and influence on not only intrauterine life of the fetus and the health of the pregnant mother but also may have significant postpartum effects on the mother’s future health and well-being. Most important of these may be the hormonal factor from the fetus that have a major impact on obstetrical outcomes and differences between male and female fetus pregnancies. The pathophysiology of some of these sex-specific differences has been well elucidated while others remain unclear. Based on a review of current literature published in English language from around the world, this review summarizes the specific differences in pregnancies and postpartum outcome based on the fetal sex.

## Methods

This is a narrative review. We searched https://www.ncbi.nlm.nih.gov/pubmed for the keywords “fetal gender” and “fetal sex” using a filter of publications in the last 10 years. Most of these articles were irrelevant to fetal gender or fetal sex and they appeared in the search result because they contain the word “fetal” only. Exclusion criteria were the effect of fetal sex or gender on neonatal outcomes and the effect of fetal sex or gender on infantile outcomes. We also included some articles that were published more than 10 years ago that were found while reviewing the references of the newer articles. We chose to cover the fetal sex effects on the following topics depending on the importance and the number of relevant articles: antenatal complications, maternal glucose tolerance, hypertensive disorders in pregnancy, preterm delivery (PTD), fetal growth, birth weight, fetal heart rate monitoring, cellular level, maternal nutrition, prenatal alcohol exposure, and Bisphenol A exposure during pregnancy.

### Antenatal Complications and Fetal Sex

A pregnancy with a male fetus has been associated with an increased risk of pregnancy complications and adverse obstetrical outcomes. A study by Sheiner et al. (1) comparing 55,891 pregnancies with a male fetus and 53,104 with a female fetus found that when the fetal sex was male, pregnancies were found to have higher rates of fetal macrosomia [OR = 2.0; 95% confidence interval (CI) 1.8–2.1; *p* < 0.001], failure to progress during the first and second stages of labor (OR = 1.2; 95% CI 1.1–1.3; *p* < 0.001 and OR = 1.4; 95% CI 1.3–1.5; *p* < 0.001, respectively), cord prolapse (OR = 1.3; 95% CI 1.1–1.6; *p* = 0.014), nuchal cord (OR = 1.2; 95% CI 1.1–1.2; *p* < 0.001), and true umbilical cord knots (OR = 1.5; 95% CI 1.3–1.7; *p* < 0.001). Cesarean section (CS) rates were found higher among pregnancies with male compared with female fetuses (8.7 vs. 7.9%; OR = 1.1; 95% CI 1.06–1.16; *p* < 0.001). After statistically controlling confounders, they have found that having a male fetus was significantly associated with non-reassuring fetal heart rate patterns (OR = 1.5; 95% CI 1.4–1.6; *p* < 0.001), low Apgar scores at 5 min (OR = 1.5; 95% CI 1.3–1.8; *p* < 0.001), and CS (OR = 1.2; 95% CI 1.2–1.3; *p* < 0.001). They concluded that male sex is considered an independent risk factor for adverse pregnancy outcome. Actually, some of the observations of this study can be interrelated, for example, it is well known that fetal macrosomia can lead to prolonged both first and second stages of labor that in turn can result in an increase in the cesarean delivery rates, but the mechanism standing behind higher rates of macrosomia in male fetuses is not clear yet. Sex hormones, fetal insulin, and genetic factors can all interact to result in higher weight of male fetuses compared to female fetuses for a given gestational age.

### Fetal Sex and Maternal Glucose Tolerance

Studies examining an association between fetal sex and abnormal maternal carbohydrate metabolism have yielded conflicting results. One study found that after adjusting for maternal parity, age, race, pre-pregnancy BMI, education, history of gestational diabetes, smoking and alcohol use, and gestational age at blood glucose measurement, a female fetus may be associated with greater maternal insulin resistance during pregnancy (2). However, Verburg et al. (3) and Sheiner et al. (1) showed a male predominance for gestational diabetes mellitus (GDM). Another study following women with singleton live-births for a median of 3.8 years in Canada (4) found that carrying a male fetus was a risk factor of GDM in the first pregnancy and also in the second pregnancy. However, they found that the likelihood of developing type 2 diabetes mellitus (T2DM) before a second pregnancy if the first pregnancy was complicated by GDM was higher if they delivered a girl. Another interesting result of that study is that recurrence of GDM was not found to be affected by fetal sex. By following up the same study population for a median of 5.5 years after delivery, they have found that compared to carrying a male fetus, having a female fetus carries a higher risk of subsequently developing T2DM (adjusted hazard ratio = 1.06, 95% CI 1.01–1.12) (5). One suggested pathophysiology is that women carrying a male fetus have poorer beta cell function as measured by the insulinogenic index divided by HOMA of insulin resistance (6). Supporting evidence came from a study from Ireland (7) that found another interesting observation of a higher insulin resistance among female fetuses with higher leptin and C-peptide concentrations in their cord blood despite weighing less at birth. These findings are consistent with the growing body of evidence suggesting that girls are intrinsically more insulin resistant than boys in both childhood and adolescence. This Irish study was a secondary analysis of a cohort of women and infants from the ROLO randomized control trial and limited by their study population which was women who were secundigravid and having previously delivered an infant weighing greater than 4,000 g excluding women who had pre-existing or previous gestational diabetes. Shields et al. (8) have also found that despite being smaller, female newborns have higher insulin, total proinsulin, and intact proinsulin concentrations than male newborns, probably because of higher intrinsic insulin resistance in girls. This study also highlighted the influence of appropriate cord blood sample collection method. They found that cord insulin concentrations in the samples taken in heparin and stored at room temperature significantly decreased, while those taken on EDTA and refrigerated remained stable for up to 48 h. On the other hand, Eder et al. (9) could not find a statistically significant difference between cord blood insulin concentrations collected from 414 male and female newborns but, by examining subcutaneous adipose tissue thickness of 15 body sites, they found that the deposition of fat in female neonates seems less affected by insulin as compared to males. This may reflect lower insulin sensitivity in females or can be related to other metabolic/endocrine factors overriding the association. High maternal blood glucose during pregnancy carries maternal and fetal risks. Studying the fetal glucose homeostasis can be an important factor in selecting patients for more strict blood sugar control to decrease the consequences on the fetus.

### Fetal Sex and Hypertensive Disorders in Pregnancy

Hypertensive disorders are major complications of pregnancy, especially preeclampsia, which is commonly associated with significant maternal and fetal morbidity and mortality. Some studies suggest that fetal sex can be a risk factor for the development of these hypertensive disorders in pregnancy. Shiozaki et al. (10) conducted their study in 125 centers in Japan from 2001 through 2005. They found that in singleton, monochorionic diamniotic (MD), and dichorionic diamniotic (DD) pregnancies, pregnant women carrying female fetuses had a significantly higher incidence of pregnancy-induced hypertension (PIH) and preeclampsia compared with those carrying male fetuses while twin pregnancies with male–female fetuses had intermediate values. Also, they found that the incidence of PIH and preeclampsia in MD twin pregnancies were similar to those in DD twin pregnancies with male–male fetuses or female–female fetuses. They concluded that female fetal sex was a risk factor for both PIH and preeclampsia while the male antigen and the increased number of major histocompatibility complex mismatches in DD twin pregnancies may not be a risk factor for PIH and preeclampsia. The pathophysiology of hypertensive disorders of pregnancy was extensively studied and multiple risk factors have been postulated for the development of hypertensive disorders of pregnancy. One of these risk factors that have been linked is high levels of beta human chorionic gonadotropin (β-hCG). A new study published in 2016 suggested that there is a female preponderance in hypertensive pregnancies and elevated β-hCG levels (11). Higher β-hCG levels and female sex of the fetus are two independent risk factors for severe preeclampsia in the early second trimester of pregnancy (11). However, a previous study conducted in Norway showed a significant correlation between fetal sex and the levels of hCG during the third trimester only, possibly because of a sex factor and a shift in production and/or in metabolism of hCG from the second to the third trimester. The researchers of that study examined hCG levels in maternal serum and amniotic fluid at different gestational ages of 130 uncomplicated pregnancies. They found that hCG levels were significantly higher in maternal serum than in amniotic fluid. At 16 weeks, they did not find any sex-related differences in the hCG levels, either in maternal blood or in amniotic fluid. At 35 weeks, the hCG levels in maternal blood were significantly higher (*p* < 0.004) compared to slightly higher amniotic fluid hCG in pregnancies with female fetuses than in those carrying male fetuses. In pregnancies with female fetuses, the hCG levels in maternal blood were significantly higher at 35 than at 16 weeks (*p* < 0.02), while in pregnancies with male fetuses, the levels were highest at 16 weeks. Because most cases of PIH arise in the third trimester, the findings in that study suggest that carrying a female fetus is a risk factor for the development of hypertensive diseases of pregnancy because of the elevated levels of maternal serum hCG in pregnancies carrying females compared to those carrying males (12). Another suggestion for this is the differences in the renin–angiotensin system during early gestation. Sykes et al. (13) examined women who later developed gestational hypertension or preeclampsia and compared with body mass index-matched controls. They found that women who subsequently developed preeclampsia or gestational hypertension had elevated levels of angiotensin-(1–7) at 15 weeks of gestation compared with women with normal pregnancies. This difference was seen only in women carrying a female fetus when subgrouping by fetal sex. Fetal sex effect on gestational hypertension can extend to treatment. In New Zealand, Gray et al. (14) have examined the effects of MgSO_4_ on vascular tone in male and female placental vessels from term and preterm deliveries. Their results demonstrate that when presented with adverse maternal environments and associated pregnancy complications, male and female fetuses may employ sex-specific survival strategies. Their data provide evidence that the placenta functions in a sex-specific manner. They found that in preterm female pregnancies, the placental bed is able to maximally relax in response to magnesium sulfate and this leads to improved fetal nutrient delivery and gas exchange in the peripartum period and subsequently to higher overall neuroprotective effects of magnesium sulfate. These sex dimorphic placental adaptations during pregnancy can explain the sex-specific differences in perinatal morbidity and mortality and the relatively high neonatal morbidity noted in male neonates.

### Fetal Sex and PTD

Previous research suggests that being pregnant with a male fetus might be considered as one of the risk factors for PTD (15–20). Wilms et al. (21) concluded that the majority of preterm labor (PTL) cases are pregnant with a male fetus and these pregnant women deliver slightly earlier, but race appears to affect this disparity. They performed a secondary analysis of a prospective cohort study including women with symptoms of PTL between 24 and 34 weeks. They had calculated the proportion of women carrying a male or female fetus at the onset of PTL. Gestational age at delivery and risk of PTD of both fetal sexes were compared and the interaction of maternal ethnicity and fetal sex on the risk of PTD was evaluated. Of the 594 included women (55% were male pregnancies), median gestational age at delivery in women carrying a male fetus was 37 5/7 weeks compared with 38 1/7 weeks in women pregnant with a female fetus. The risk of PTD did not differ significantly. In Caucasians, they did find an increased risk of PTD before 37 weeks in women pregnant with a male fetus [OR 1.9 (95% CI 1.2–3.0)]. A retrospective population-based study of more than half a million live-births for 20 years has been recently published and showed male predominance for PTB (20–24 weeks: RR 1.351, 95% CI 1.274–1.4450), spontaneous PTB (25–29 weeks: RR 1.118, 95% CI 1.044–1.197%) but female predominance for iatrogenic PTB (25–29 weeks: RR M/F 0.857, 95% CI 0.780–0.941) (3). Regarding twin pregnancies, a retrospective population-based cohort study using the 1995–1997 registration twin data in the United States (148,234 live-birth twin pairs) divided twin pairs into 3 groups: male–male, female–female, and opposite sex. They used three different cutoff values of preterm birth: less than 28, 32, and 36 weeks of gestation and compared the preterm birth rates among these three study groups, and then adjusted risk ratios (relative risk) by multiple logistic regression. They found that the male–male twin pairs group had the highest preterm birth rate, the female–female twin pairs were intermediate, and the opposite sex twin pairs group had the lowest rate. Adjustment for important confounding factors or excluding twin pairs born to mothers who had an induction of labor or a cesarean delivery with medical complications did not change their results. The adjusted relative risks (95% CI) of the three cutoff values were 1.19 (1.11–1.27), 1.21 (1.16–1.26), and 1.09 (1.07–1.11), respectively, for male–male twins compared with the opposite sex twins. They concluded that male sex is associated with increased risk of preterm births in twin pregnancy (22). The relatively greater weight of male fetuses compared to female fetuses for a given gestational in addition to the increased vulnerability of infection of women carrying a male fetus can explain at least in part the higher incidence of PTD in male fetus pregnancies (15).

### Fetal Sex and Fetal Growth

Sex differences in fetal growth have been recognized in many studies which can be established as early as 15 weeks of gestation (23–25). In a recent study, the applications of these differences were used to customize fetal growth charts based on fetal sex (26). In this study, investigators constructed customized biometric growth charts for fetal sex, parental, and obstetrical characteristics using quantile regression. In this large multicenter cross-sectional study, 8,070 ultrasonographic examinations from low risk singleton pregnancies between 16 and 40 weeks of gestation were examined. They obtained the following fetal measurements: biparietal diameter (BPD), head circumference (HC), abdominal circumference (AC), and femur length (FL). Quantile regression was used to examine the impact of fetal sex across the biometric percentiles of those fetal measurements considered together with parents’ height, weight, parity, and race. They found that fetal sex was a significant covariate for BPD, HC, and AC with higher values for male fetuses and minimal differences for FL were found among sexes. Other confounders like parity, paternal/maternal height, and maternal race and weight were significantly related to these fetal biometric parameters but they considered independently from fetal sex. The use of sex-specific fetal growth charts may offer the advantage to more accurately detect abnormal fetal growth. Preeclamptic pregnancies have higher serum leptin levels than normal pregnancies (27), and leptin levels were found to be higher in the amniotic fluid in IUGR female fetuses than in IUGR male fetuses (28). Higher incidence of preeclampsia in women carrying female fetuses is one explanation of lower weight of female fetuses.

### Fetal Sex and Birth Weight

Muhihi et al. (29) analyzed birth outcome data from singleton infants, who were enrolled in a large randomized, double-blind, placebo-controlled trial of neonatal vitamin A supplementation conducted in Tanzania. Among 19,269 singleton Tanzanian newborns included in this analysis, 68.3% were term deliveries and appropriate for gestational age (AGA), 15.8% were also term deliveries but small for gestational age (SGA) (defined as birth weight less than 10th percentile), 15.5% were preterm deliveries and AGA, and 0.3% were preterm and SGA. In their multivariate analyses, they have found that male fetus was a significant risk factors for term-SGA (*p* < 0.05).

### Fetal Sex and Fetal Heart Rate (FHR) Monitoring

Kim et al. (30) examined the antepartum FHR indices, dynamics, complexity, and reactivity to the non-stress test and vibroacoustic-stimulation test of a total of 3,835 (1,849 females and 1,986 males) singletons who delivered at term without maternal and fetal complications. They suggested that the cardiovascular system of female fetuses matures earlier than that of males because female fetuses exhibited greater heart rate dynamics in early gestational periods but male fetuses undergo a compensatory period of rapid changes to catch up with females at term. That is why interpretation of electronic fetal monitoring (EFM) patterns may need to take into account factors such as fetal sex. Few studies have examined the influence of fetal sex on cardiotogoraphic (CTG) parameters, and most of them have failed to demonstrate significant sex differences in the antepartum period (31, 32). Dawes et al. (33) found that fetal heart rate is significantly higher in female fetuses than male fetuses but the sex differences in fetal heart rate was less than 6–7 h before delivery and in the first stage of labor, and no difference was noted before the onset of labor. DiPietro et al. (34) found a higher heart rate variability throughout gestation in males. Bernardes et al. (35) have suggested sex differences in the activity of autonomic nervous system by reporting significantly more linear and significantly less complex fetal heart rate activity in male than female fetuses. Amorim-Costa et al. (36) published a large study where they analyzed fetal heart tracings for a median duration of 29.4 min per fetus for 9,701 fetuses. This study provides reference values for CTG parameters throughout pregnancy. In this study, sex differences were clearly demonstrated in fetal life, and percentile curves were constructed separately for male and female fetuses. Another study examining 2,639 deliveries, where 1,400 (53%) were male. In this study, male fetuses were found to have a higher number of decelerations (*p* < 0.003) in addition to increased total deceleration area [adjusted odds ratio (aOR): 1.11, 95% CI: 1.04–1.18]. Male fetuses were also found to be at increased risk for both repetitive variable decelerations (aOR: 1.24, 95% CI: 1.05–1.47) and prolonged decelerations (aOR: 1.21, 95% CI: 1.03–1.42). This study suggests that there may be significant sex differences in EFM patterns at term among pregnancies without evidence of academia (37). One explanation of these differences is that brain anatomy and the chemistry of neuronal transmission is affected by the protocadherin gene expression in the brain. This expression is thought to be influenced by genes on the Y chromosome (38). Also, preterm females were found to have higher levels of catecholamines in blood than preterm males after exposure to asphyxia (39), this mechanism can explain the higher heart rates and better outcome of female infants compared to male infants. These observations were not interpreted into the real clinical practice guideline yet, but it is important to put in mind these fetal sex differences when analyzing the fetal heart rate strip.

### Maternal Nutrition and Fetal Sex

It is clear that the nutritional status of the pregnant mothers affects their fetuses and the outcome of their pregnancy, but is there any role of the fetal sex in this? Cogollos et al. (40) performed their study aiming to determine whether developmental patterns, adiposity level, and fatty-acid composition of fetuses exposed to maternal malnutrition are driven by their sex or their genotype, or both, because these two factors may influence the adaptive response of the fetus to the intrauterine environment independently of the maternal genotype. In this animal study, they obtained four different subsets of fetuses by using a single maternal genotype [purebred Iberian (IB) sows], which was inseminated with heterospermic semen [obtained by mixing semen from IB and large white (LW) boars], the four subsets are male and female, purebred (IB × IB), and crossbred (IB × LW) in IB purebred sows. Better adaptive response was observed in the female offspring and this was modulated by their genotype also. Females were found to prioritize the growth of vital organs (liver, brain, kidneys, lungs, and intestine) at the expense of bone and muscle when faced with prenatal undernutrition. Higher availability of essential fatty acids in the female sex and IB genotype than in their male and crossbred fetuses counterparts was noted. This interesting study showed how the fetal sex and the genotype can affect the metabolic pathway and prenatal development. Mandò et al. (41) performed another study in Italy, they prospectively enrolled 696 women: 537 normal weight (NW) (18 ≤ BMI < 25 kg/m^2^), 112 overweight (OW) (25 ≤ BMI < 30 kg/m^2^), 47 obese (OB) (BMI ≥ 30 kg/m^2^) with singleton uncomplicated pregnancies at term delivery. They collected the following data gestational age, maternal (age, height, pre-pregnancy BMI, gestational weight gain, hemoglobin, hematocrit, and glycemia), fetal (weight, length, ponderal index, cranial circumference), and placental (weight and diameters). Placental area, thickness, and efficiency (fetal/placental weight ratio, F/P) were calculated. In the total population, they have found a significant interaction effect between maternal BMI and fetal sex on placental weight and efficiency as differences in placental parameters were present only in female offspring. They have reported a difference in placental adaptation depending on fetal sex, with significant changes only in female fetuses. This is important because it can explain why female fetuses do better than males in terms of survival. So, we, as health-care providers, may customize our counseling according to maternal body habitus and sex of the fetus in clinical settings. Another study conducted in the United States (42) has divided the study cohort into 192 normal weight (BMI = 20–24.9) pregnant mothers and 231 obese (BMI ≥ 30) pregnant mothers with the assessment of demographic, obstetrical, and neonatal variables. The researchers of this study examined the placental histopathology focusing on inflammatory markers. These placental characteristics were placental disc weight >90th percentile, decreased placental efficiency, chronic villitis (CV), fetal thrombosis, and normoblastemia. Fetal thrombosis and higher rates of CV were observed in female fetuses of obese mothers. However, the final grade and extent of CV was significantly associated with obesity and BMI, but not fetal sex. They have shown for the first time that the effect of maternal obesity on placental inflammation is independent of hypertension and diabetes, but significantly affected by fetal sex.

### Fetal Sex and Alcohol and Bisphenol A Exposure during Pregnancy

The development of the fetal central nervous system (CNS) is affected by alcohol consumption during pregnancy. The hippocampus appears to be highly affected especially if alcohol was maternally abused during specific periods in pregnancy. The effect of alcohol on the CNS can be at the signaling and synaptic level through interference with receptor proteins and hormones. Male and female fetuses can be differentially affected by this toxic exposure during pregnancy possibly through hippocampal synaptic plasticity. Some sex differences have been noted in examining the effects of prenatal and/or early postnatal alcohol exposure. Prenatal and/or early postnatal alcohol exposure has been shown to reduce hippocampal cell survival in male offspring only, and it may lead to a higher basal estrogen levels that can play a significant role in synaptic plasticity (43).

Another recently emerging topic is the Bisphenol A exposure during pregnancy. Veiga-Lopez et al. (44) published a study in 2015, they suggested that higher unconjugated BPA (uBPA) exposure levels during first trimester and term are associated with sex-specific reduction in birth weight and increase in gestational length, respectively. When combining both sexes, a twofold increase in first trimester uBPA was associated with 55-g less birth weight while a 183-g less birth weight was noted in only female pregnancies. Gestational length increased 0.7 days in association with a twofold increase in maternal term uBPA when combining both sexes and 1.1 days if only females were included. These sex differences can be attributed to either enzymatic and metabolic differences between male and female fetuses or differences in the levels of maturation of such chemical processes. Knowing these differences may encourage more detailed biochemical and pathophysiological studies in this field that can yield more clinically important data.

### Fetal Sex and Cellular Level

Measuring the telomere length is an evolving field that has attracted attention recently, it has been linked with some obstetrical and problems later in life (such as IUGR and cancers, respectively) (45, 46). A recently published study involved analysis of cord blood telomere length of 54 infants. Female sex was associated with longer telomere length by ~350 bp leading to the conclusion that male infants have a shorter telomere length at birth, but whether this can predict any worse outcome in males than in females is not yet clear and more studies are required to figure out this association (47). Different body organs of male and female fetuses may display distinct antioxidant pathways. In a study from France (48), brain, lung, liver, kidney, and skeletal muscles of female and male fetuses of sheep twin pregnancies at day 65 of gestation were examined for the activity of total superoxide dismutase (SOD), SOD1, SOD2, glutathione peroxidase, glutathione reductase (GR), and catalase, the content of total glutathione, reduced glutathione (GSH), and oxidized glutathione (GSSG). In this study, male brain has greater total SOD and SOD1 activities than female brain. Female liver has been found to have greater SOD2 activity than male liver. Male liver had greater GR activity than female liver. Male liver had higher total GSH and GSSG content than female liver. Male skeletal muscles had higher total GSH, GSH, and GSSG content than female skeletal muscles. The brain and liver of females have higher lipid peroxidation activity (as measured by melondialdehyde tissue content) than male brain and liver. According to the authors of this study, this was the first report of a sex dimorphism for fetal organ antioxidative pathways. Because of the well-known oxidative damage to fetal tissues and organs at critical developmental windows represents a common underlying mechanism connecting both fetal programming and subsequent health outcomes (49), this study has suggested that such sex-specific dimorphic responses of fetal cells to early exposure to oxidative stress might be involved in the sex-related difference in fetal development that may have long-term influence on offspring (48). Researchers have to be aware of these sex differences in cell biology when they conduct their investigations. It is known that male fetuses and neonates show a relatively higher immune vulnerability compared to females (50). This can result in a higher risk of perinatal infections and can be partially explained by the differential modulation of vitamin D metabolism [slower immunological development is also postulated as a risk factor for the increased risk of fetal membranes and amniotic fluid infections in male fetuses compared to female fetuses (51)]. Calcitriol, which is the most active form of vitamin D, regulates immune responses and also transcriptionally induces the antimicrobial peptide cathelicidin in the human placenta (52, 53). CYP27B1 and CYP24A1 are cytochromes involved in calcitriol synthesis and degradation, respectively, so calcitriol availability depends on expression of these two cytochromes (54). Olmos-Ortiz et al. (55) examined placentas and umbilical vein cord blood from term uncomplicated pregnancies. They have showed that testosterone significantly inhibited CYP27B1 while stimulated CYP24A1 gene expression in cultured trophoblasts. Reduced basal cathelicidin gene expression was found in male placental cotyledons and this results in lower cathelicidin levels in the cord blood compared to females. Their results suggest that male placentas produce less cathelicidin due to decreased calcitriol bioavailability and this may partially explain the higher risk of infection in male fetuses and neonates.

### Stillbirth Risks and Fetal Sex

The effects of fetal sex on stillbirth rates have been reported over a long period of time. Sex difference in the birth rate was initially reported as observational finding and later researchers sought to correlate this finding with pathophysiological changes between males and females placentae and the feto-placental hormonal milieu. Mondal et al. (56) conducted one of the largest systematic reviews in this field. After reviewing more than 30 million births from studies in various parts of the world, they found that stillborn males exceed stillborn females in the crude mean rate (stillbirths/1,000 total births). They also found that the stillbirth risk is 10% higher in male fetuses. Importantly, this study found that these risks to male fetuses do not differ whether definition of stillbirth was at 20 or 28 weeks. An explanation of this higher risk of stillbirth in male fetuses has been attributed to the difference in the metabolism and susceptibility to nutritional factors between male and female placentae. Contrary findings have been reported from studies in India and China that found the stillbirth risk to be either equal in both sexes (57) or higher in female pregnancies (58); but cultural factors that favor a live born male in these countries should caution us in interpreting these results (56). Further studies are needed to establish a clear causal relationship between fetal sex and stillbirth rate.

### Should Care of Pregnant Women Differ Based on Fetal Sex?

Even though there are differences in pregnancy outcomes based on fetal sex in singleton pregnancies, the answer to the above question is still not clear. Some obstetricians feel that care of pregnant women has to be individualized according to many factors including fetal sex (59). Others believe that fetal sex is never a determinant factor in individualization of maternal care. However, this individualization of care of pregnant women is more relevant to screening of obstetrical conditions and complications but the implications for treatment are not well studied. With increased use of cell free fetal DNA testing, fetal sex is available to most obstetricians by the end of the first trimester. Given the push for individualized care, it is quite likely that in the near future obstetricians may apply more sophisticated and targeted screening measures, depending on fetal sex, to improve outcomes in mothers and babies.

## Summary

This review has attempted to summarize the sex-specific differences in pregnancy outcomes. We have also tried to explain maternal–placental–fetal interactions based on the temporal sequence of effects starting with conception and early gestation, going through the mid and last trimesters and concluding with post-pregnancy and general effects that have been described in the literature (summarized in Table 1).

**Table 1:** Effects of maternal interactions with male and female fetuses during and after pregnancy.

Time	Maternal interaction with male fetus	Maternal interaction with female fetus	Reference
Early pregnancy	Maternal alcohol consumption—reduced hippocampal cell survival in male fetus	Maternal alcohol consumption—no effect on hippocampal cells in female fetus	Fontaine et al. (43)
	Maternal unconjugated BPA (uBPA) exposure—minimal effect on weight	Maternal uBPA exposure—higher decrease in birth weight	Veiga-Lopez et al. (44)

Mid-pregnancy	Male fetus—no effect on maternal Angiotensin-(1–7) noted in relation to pregnancy-induced hypertension (PIH)	Female fetus—elevated maternal levels of angiotensin-(1–7) in relation to PIH	Sykes et al. (13)
	Delayed CVS maturity	Earlier CVS maturity	Kim et al. (30)
	^a^Antioxidant pathway—male fetus: Greater total superoxide dismutase (SOD) and SOD1 activities in brain Greater GR activity in the liver Higher total GSH and GSSG content in the liver Higher total GSH, GSH, and GSSG content in skeletal muscles	^a^Antioxidant pathway—female fetus: Greater SOD2 activity in the liver Higher lipid peroxidation activity in brain and liver	Al-Gubory and Garrel (48)

Late pregnancy	Higher rate of macrosomia	Lower rate of macrosomia	Sheiner et al. (1)
	Lower maternal insulin resistance	Higher maternal insulin resistance	Xiao et al. (2)
	More incidence of gestational diabetes mellitus (GDM) in current pregnancy	Less incidence of GDM in current pregnancy	Sheiner et al. (1), Verburg et al. (3), Retnakaran and Shah (4)
	Poorer maternal beta cell function	Better maternal beta cell function	Retnakaran et al. (6)
	Lower fetal insulin resistance and lower cord blood leptin and C-peptide	Higher fetal insulin resistance and higher cord blood leptin and C-peptide	Walsh et al. (7)
	Lower incidence of PIH and preeclampsia	Higher incidence of PIH and preeclampsia	Shiozaki et al. (10), Zheng et al. (11)
	Lower maternal hCG levels	Higher maternal hCG levels	Steier et al. (12)
	Higher measurements of biparietal diameter (BPD), head circumference (HC), and abdominal circumference (AC)	Lower measurements of BPD, HC, and AC	Rizzo et al. (26)
	Higher incidence of term- small for gestational age (SGA)	Lower incidence of term-SGA	Muhihi et al. (29)
	Slower HR	Faster HR	Dawes et al. (33)
	More linear and complex fetal heart rate (FHR) activity	Less linear and complex FHR activity	Bernardes et al. (35)
	Less increase in gestational length on uBPA exposure	More increase in gestational length on uBPA exposure	Veiga-Lopez et al. (44)
	Higher stillbirth rates	Lower stillbirth rates	Mondal et al. (56)

Labor	Higher rate of failure to progress in first stage	Lower rate of failure to progress in first stage	Sheiner et al. (1)
	Higher rate of failure to progress in second stage	Lower rate of failure to progress in second stage	Sheiner et al. (1)
	Higher rate of non-reassuring fetal heart rate patterns	Lower rate of non-reassuring fetal heart rate patterns	Sheiner et al. (1)
	Lower levels of cord blood insulin, total proinsulin, and intact proinsulin concentrations	Higher levels of cord blood insulin, total proinsulin, and intact proinsulin concentrations	Shields et al. (8)
	Higher number of decelerations, increased total deceleration area, increased risk for both repetitive variable decelerations and prolonged decelerations	Lower number of decelerations, decreased total deceleration area, decreased risk for both repetitive variable decelerations and prolonged decelerations	Porter et al. (37)

Delivery	Higher rate of Nuchal cord	Lower rate of Nuchal cord	Sheiner et al. (1)
	Higher rate of true umbilical cord knots	Lower rate of true umbilical cord knots	Sheiner et al. (1)
	Higher cesarean section (CS) rate	Lower CS rate	Sheiner et al. (1)
	Higher rate of low Apgar scores at 5 min	Lower rate of low Apgar scores at 5 min	Sheiner et al. (1)
	Higher PTD rate	Lower PTD rate	Verburg et al. (3), McGregor et al. (15), Cooperstock and Campbell (16), Astolfi and Zonta (17), Zeitlin et al. (18), Vatten and Skjaerven (19), Challis et al. (20), Wilms et al. (21)
	Higher male–male twin PTD rate	Lower male–male twin PTD rate	Tan et al. (22)
	Lower rate of iatrogenic PTD	Higher rate of iatrogenic PTD	Verburg et al. (3)
	Higher rate of cord prolapse	Lower rate of cord prolapse	Sheiner et al. (1)

Placenta	Minimal relaxation in response to MgSO_4_	Maximal relaxation in response to MgSO_4_	Gray et al. (14)
	Inhibited CYP27B1 and stimulated CYP24A1 gene expression by male fetus androgens	No effect	Olmos-Ortiz et al. (55)
	Reduced basal cathelicidin gene expression leading to lower cathelicidin levels in the cord blood	Higher basal cathelicidin gene expression and higher cathelicidin levels in the cord blood	Olmos-Ortiz et al. (55)
	Maternal BMI-No effect on placental weight and efficiency	Maternal BMI-Observed effect on placental weight and efficiency	Mandò et al. (41)
	Lower rate fetal thrombosis and chronic villitis (CV) in obese mothers	Higher rate fetal thrombosis and CV in obese mothers	Leon-Garcia et al. (42)

Postpartum–maternal	Lower risk of GDM in second (next) pregnancy	Higher risk of GDM in second (next) pregnancy	Retnakaran and Shah (4)
	Lower risk of developing type 2 diabetes mellitus (T2DM) before a second (next) pregnancy	Higher risk of developing T2DM before a second (next) pregnancy	Retnakaran and Shah (4)
	Lower risk of developing T2DM in 5.5 years follow-up	Higher risk of developing T2DM in 5.5 years follow-up	Retnakaran and Shah (5)

General	Higher heart rate variability throughout gestation	Lower heart rate variability throughout gestation	DiPietro et al. (34)
	^a^Maternal malnutrition-Less adaptive response and lower availability of essential fatty acids	^a^Maternal malnutrition-Better adaptive response through prioritization of growth of vital organs (liver, brain, kidneys, lungs, and intestine) at the expense of bone and muscle and higher availability of essential fatty acids	Cogollos et al. (40)
	Higher immune vulnerability	Lower immune vulnerability	Klein (50)
	Shorter telomere length	Longer telomere length	Wojcicki et al. (47)

*^a^Sheep data*.

*CVS, cardiovascular system*.

## Conclusion

There may be differences in pregnancy and neonatal outcomes based on the sex of the fetus in a pregnancy. Hypertension, diabetes, and PTD may be more predictable depending on fetal sex, and this may require more prophylactic measures to be considered. Interpretation of the cardiotocograph strips and fetal growth charts can be modified in the future according to fetal sex but this may require more sophisticated and large studies before reaching such point. We also suggest more studies to further define the pathophysiology and impact of these sex differences on fetuses, mothers, and neonates.

## Author Contributions

MA was involved in the concept and design of the review. He reviewed all the references, designed the table and figure, and wrote the abstract and provisional manuscript and the final manuscript. YF reviewed all the references, reviewed the table and figure, and wrote the abstract and provisional manuscript and the final manuscript.

## Conflict of Interest Statement

The authors declare that the research was conducted in the absence of any commercial or financial relationships that could be construed as a potential conflict of interest.
